# The Influence of Processing Parameters on the Mitigation of Deoxynivalenol during Industrial Baking

**DOI:** 10.3390/toxins11060317

**Published:** 2019-06-04

**Authors:** David Stadler, Francesca Lambertini, Lydia Woelflingseder, Heidi Schwartz-Zimmermann, Doris Marko, Michele Suman, Franz Berthiller, Rudolf Krska

**Affiliations:** 1 Institute of Bioanalytics and Agro-Metabolomics, Department of Agrobiotechnology (IFA-Tulln), University of Natural Resources and Life Sciences, Vienna (BOKU), Konrad-Lorenz-Str. 20, 3430 Tulln, Austria; david.stadler@boku.ac.at (D.S.); heidi.schwartz@boku.ac.at (H.S.-Z.); rudolf.krska@boku.ac.at or r.krska@qub.ac.uk (R.K.); 2 Barilla G. R. F.lli SpA, Advanced Laboratory Research, via Mantova 166, 43122 Parma, Italy; francesca.lambertini@barilla.com (F.L.); michele.suman@barilla.com (M.S.); 3 Department of Food Chemistry and Toxicology, University of Vienna, Währingerstraße 38, 1090 Vienna, Austria; lydia.woelflingseder@univie.ac.at (L.W.); doris.marko@univie.ac.at (D.M.); 4 Institute for Global Food Security, School of Biological Sciences, Queens University Belfast, University Road, Belfast BT7 1NN, Northern Ireland, UK

**Keywords:** mycotoxins, trichothecenes, thermal degradation, decontamination, mass spectrometry, food processing, detoxification, design of experiment, LC-MS/MS

## Abstract

Deoxynivalenol (DON), a frequent contaminant of flour, can be partially degraded by baking. It is not clear: (i) How the choice of processing parameter (i.e., ingredients, leavening, and baking conditions) affects DON degradation and thus (ii) how much DON can be degraded during the large-scale industrial production of bakery products. Crackers, biscuits, and bread were produced from naturally contaminated flour using different processing conditions. DON degradation during baking was quantified with the most accurate analytical methodology available for this Fusarium toxin, which is based on liquid chromatography tandem mass spectrometry. Depending on the processing conditions, 0–21%, 4–16%, and 2–5% DON were degraded during the production of crackers, biscuits, and bread, respectively. A higher NaHCO_3_ concentration, baking time, and baking temperature caused higher DON degradation. NH_4_HCO_3_, yeast, vinegar, and sucrose concentration as well as leavening time did not enhance DON degradation. In vitro cell viability assays confirmed that the major degradation product isoDON is considerably less toxic than DON. This proves for the first time that large-scale industrial baking results in partial detoxification of DON, which can be enhanced by process management.

## 1. Introduction

Deoxynivalenol (DON) is along with aflatoxins, fumonisins, ochratoxin A, and zearalenone, one of the main mycotoxins of significance for human disease [1]. DON frequently contaminates cereals and cereal-based products [2]. The population of the European Union (EU) is frequently exposed to DON mainly due to the consumption of bread and other bakery wares [2]. Therefore, the European Commission (EC) has set maximum levels for DON in milling products and bread/bakery wares of 750 µg/kg and 500 µg/kg, respectively [3]. This legislation follows the rationale that it is feasible to reduce DON from flour during the production of bakery products by 33%. This assumption is supported by the recent report on the risk associated with DON in food by the European Food Safety Authority (EFSA) [2]. From 2007 to 2014, EFSA gathered occurrence data for the concentration of DON present in different food groups. For milling products, bread and rolls, and fine bakery wares, 4609, 2837, and 975 data points, respectively, were collected from the EU member states. The mean and 95th percentile concentrations of DON were 20–30% lower in bread and bakery wares (bread and rolls, fine bakery wares) than in flour (milling products).

During baking, DON was found to degrade mainly to isoDON, but also to norDON B and norDON C [4,5,6]. norDON B and norDON C were found to be considerably less cytotoxic than DON [5]. isoDON was found to have much lower inhibitory potency on the ribosome than DON, which is strongly indicative of its overall lower toxicity [6].

The reduction of the DON concentration during baking can be due to formulation (i.e., the dilution effect due to mixing contaminated flour with non-contaminated ingredients) or due to degradation of DON. The reduction due to formulation can be easily calculated from the percentage of flour of the finished bakery product, assuming no additional sources of DON. The extent of the DON degradation during baking, however, was reported to be very variable [2,7,8]. This high variation can be explained by errors in the calculation of the amount of DON that was degraded (e.g., by not considering the dilution with other ingredients) and by not performing baking trials with a suitable number of replicates. Recently, we identified all degradation products of DON, formed during baking of crackers, biscuits, and bread, using a stable isotope labelling approach combined with high resolution mass spectrometry analysis. After quantifying DON and all of its formed degradation products, we were able to provide the first comprehensive mass balance for those commodities [6].

In this current study, DON degradation during the production of crackers, biscuits, and bread from flour fortified with DON was determined to be 6%, 5%, and 2%, respectively, based on the increase of degradation products. As this study was aimed at the elucidation of the degradation products that are formed during baking, the experiments were carried out under standard baking conditions. As a step forward, the influence of the processing conditions should be evaluated. Furthermore, experiments with naturally contaminated flour need to be conducted in order to reproduce the actual industrial starting conditions in place during the large-scale production of bakery products.

The influence of different processing parameters on DON degradation during the production of crackers, biscuits, bread, and rusks was determined in previous studies [9,10,11,12]. Statistical evaluation of the DON concentration showed that baking temperature, baking time, and the concentration of the raising agent NaHCO_3_ influence the amount of DON in the final products. However, the statistical models as well as the determination of the DON degradation may have suffered from the high analytical variability of the determination of the DON concentration.

We hypothesized that DON degradation during industrial baking is influenced by the choice of processing parameter (i.e., ingredients, leavening, and baking conditions). Hence, the objectives of this study were to: (i) Produce bakery products (crackers, biscuits, and bread) from naturally contaminated flour using different processing conditions, (ii) determine DON degradation by LC-MS/MS based quantification of the DON degradation products and iii) develop a statistical model which shows the influence of the processing parameters on the DON degradation. Due to the comprehensive baking trials in combination with the highly sensitive LC-MS/MS analysis and the consideration of all formed degradation products, this study presents the most accurate report on the effect of industrial baking on DON degradation to date. Moreover, a first characterization of the cytotoxicity of isoDON, compared to DON, is presented.

## 2. Results

**Figure 1:**
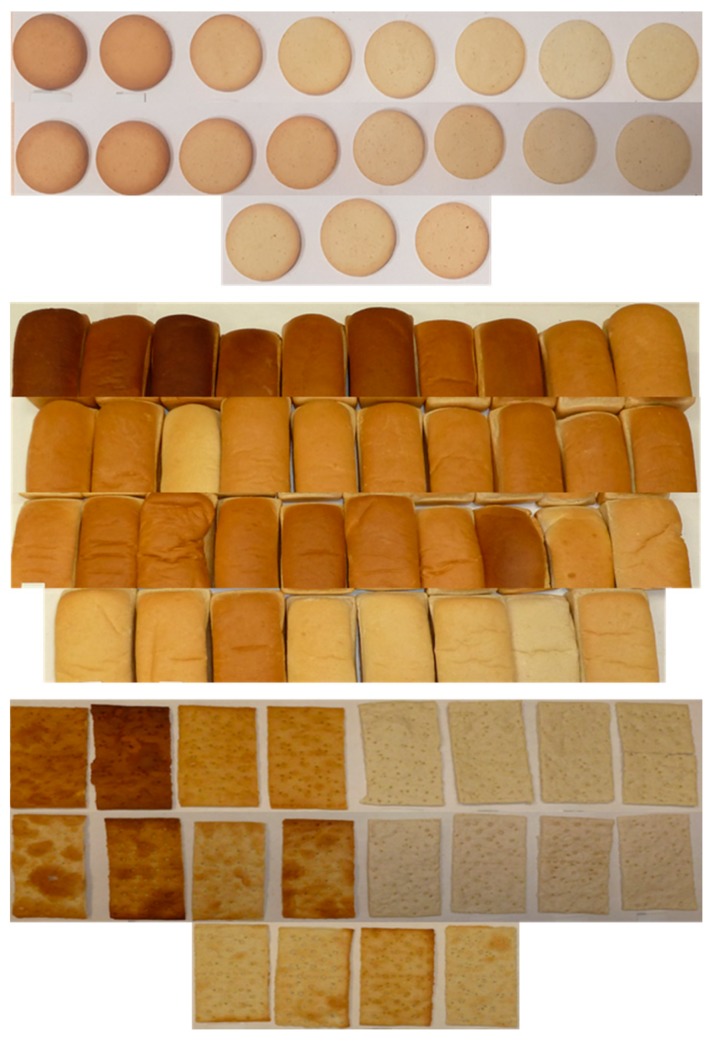
Display of the influence of baking conditions on the appearance of biscuits (top), bread (middle) and crackers (bottom).

To study the main processing factors affecting the mitigation of DON during industrial baking, crackers, biscuits, and bread were chosen as representative commodities of the main bakery categories produced with very different technologies. A Design of Experiments (DoE) approach was applied to set up the experimental trials. In agreement with results previously obtained by our research group [9,10,12], the main technological parameters monitored were: (i) Baking conditions (i.e., time and temperature), (ii) pH modifying agents (NaHCO_3_, NH_4_HCO_3_, and other minor ingredients) and (iii) leavening conditions (time, temperature, presence, and type of leavening agents). Each factor was modified within a range according to technological feasibility. Some conditions stressed the process over the organoleptic acceptability of the final product. These processing conditions were chosen to promote significant changes of the DON concentration in order to understand the impact of the applied processing conditions. In the present work, all the food commodities were produced at a pilot plant level. As a starting point of the DoE approach, the same processing parameters as those on common industrial processing lines were chosen. Figure 1 exemplifies the influence of the baking conditions on the final appearance of biscuits, bread and crackers.

### 2.1. Incluence of Processing Parameters on DON Degradation during the Production of Bakery Products

**Figure 2:**
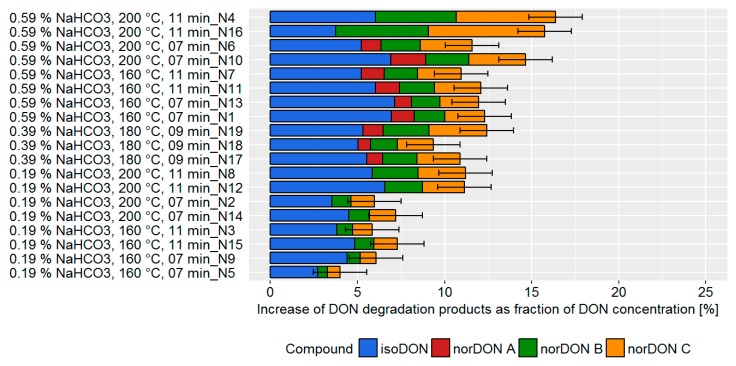
Increase of the deoxynivalenol (DON) degradation products isoDON and norDONs A-C during the production of biscuits using different processing conditions. The increase was determined as a ratio of the molal concentration of the DON degradation products to the molal concentration of DON resulting from the natural contamination of the flour. The experimental trials were listed according to the NaHCO_3_ concentration, baking temperature, and baking time. Error bars represent the process standard deviation.

**Figure 3:**
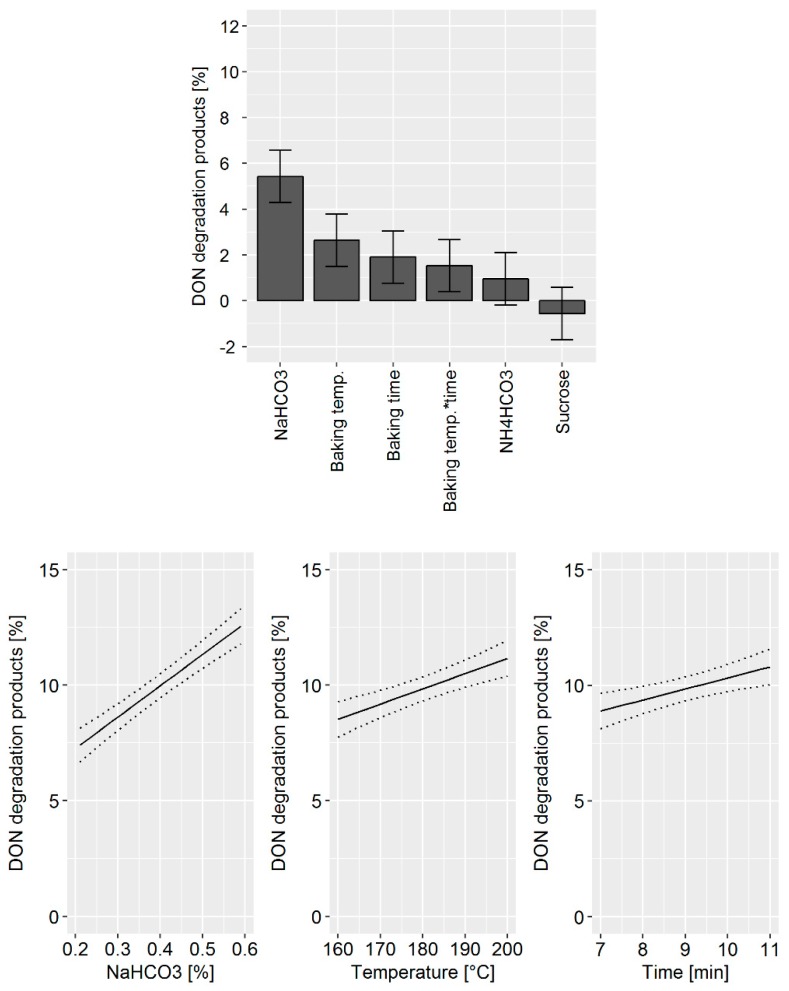
Top: Effects plot, which shows the change of the sum of the DON degradation products when a processing factor is varied from its lowest to its highest value and all other factors are kept at their averages, which was obtained for the design of the experiment (DoE) data set in pilot-scale biscuit making experiments. Error bars represent the confidence interval corresponding to a 95% confidence level. “*” between two processing factors indicates a synergistic effect, which cannot be explained solely by addition of degradation caused by the two parameters individually. Bottom: Predictive factor effect plots, which show the influence of the following processing parameters on the deoxynivalenol (DON) degradation during the biscuit production: (i) NaHCO_3_ (left), (ii) baking temperature (middle), and (iii) baking time (right). The dotted lines represent the confidence interval corresponding to a 95% confidence level.

**Figure 4:**
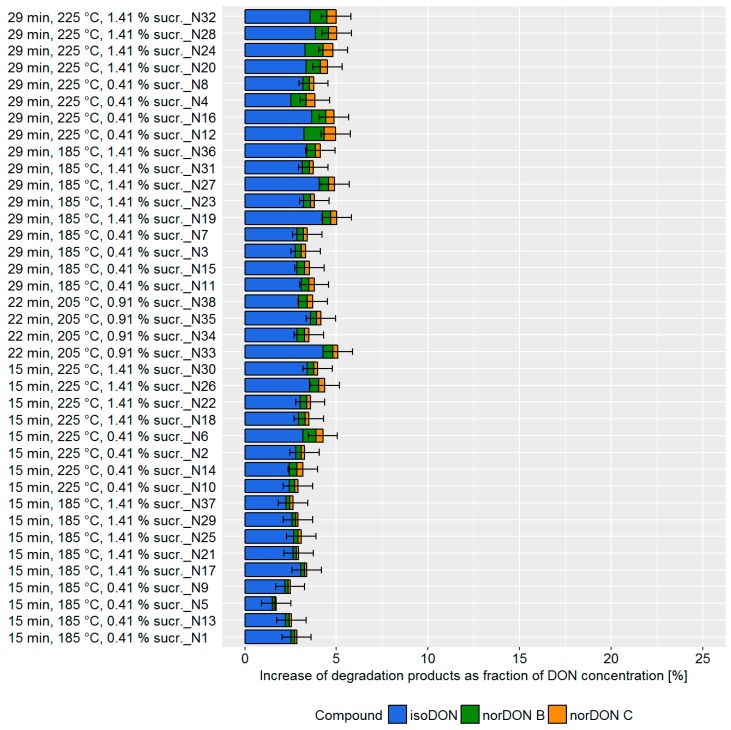
Increase of the deoxynivalenol (DON) degradation products isoDON and norDONs B-C during the production of bread using different processing conditions. The increase was determined as a ratio of the molal concentration of the DON degradation products to the molal concentration of DON resulting from the natural contamination of the flour. The experimental trials were listed according to baking temperature, baking time, and sucrose concentration. Error bars represent the process standard deviation.

**Figure 5:**
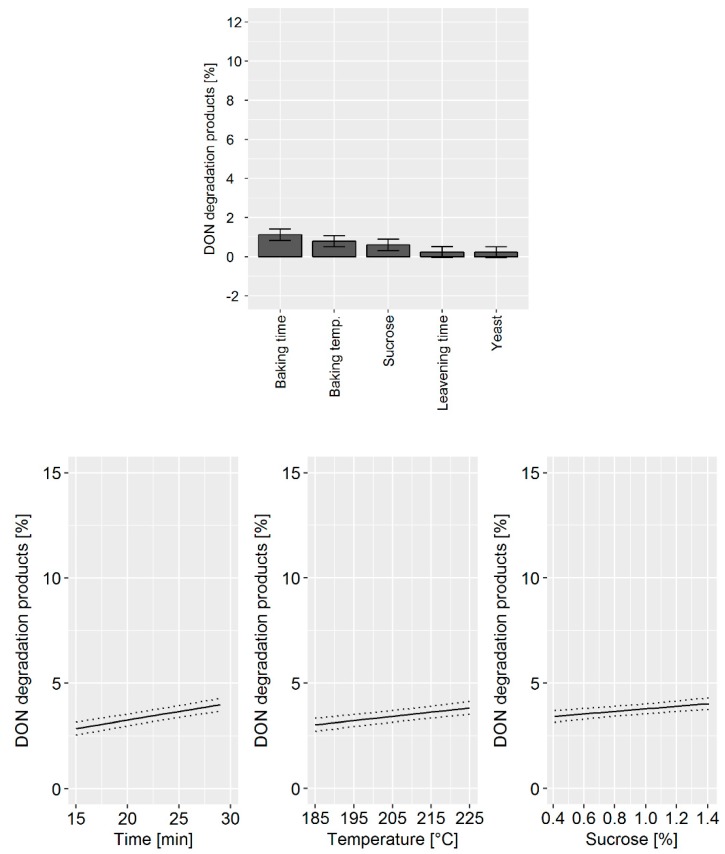
Top: Effects plot, which shows the change of the sum of the DON degradation products when a processing factor is varied from its lowest to its highest value and all other factors are kept at their averages, which was obtained for the design of the experiment (DoE) data set in pilot-scale bread making experiments. Error bars represent the confidence interval corresponding to a 95% confidence level. Bottom: Predictive factor effect plots, which show the influence of the following processing parameters on the deoxynivalenol (DON) degradation during the biscuit production: (i) Baking time (left), (ii) baking temperature (middle), and (iii) sucrose concentration (right). The dotted lines represent the confidence interval corresponding to a 95% confidence level.

**Figure 6:**
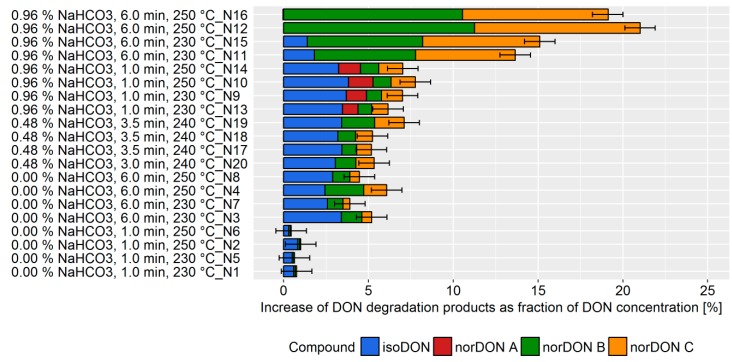
Increase of the deoxynivalenol (DON) degradation products isoDON and norDONs A-C during the production of crackers using different processing conditions. The increase was determined as a ratio of the molal concentration of the DON degradation products to the molal concentration of DON resulting from the natural contamination of the flour. The experimental trials were listed according to the NaHCO_3_ concentration, baking time, and baking temperature. Error bars represent the process standard deviation.

**Figure 7:**
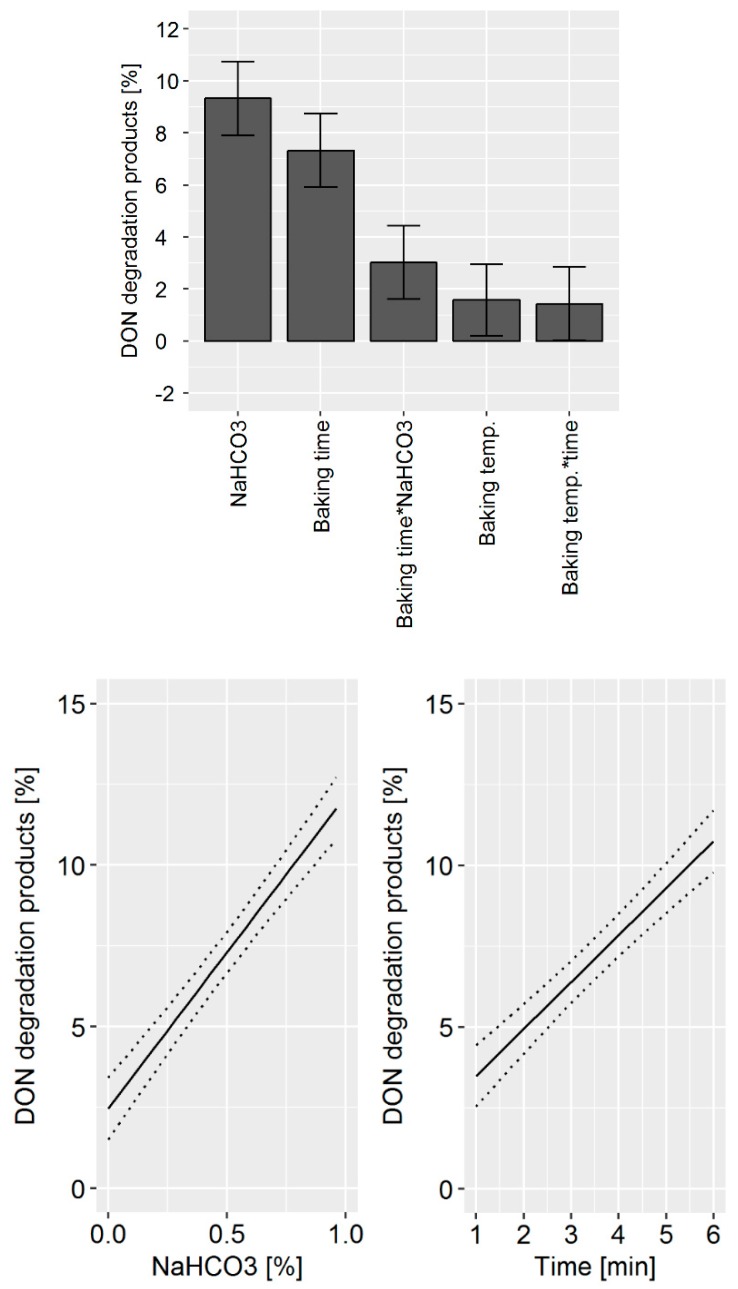
Top: Effects plot, which shows the change of the sum of the DON degradation products when a processing factor is varied from its lowest to its highest value and all other factors are kept at their averages, which was obtained for the design of the experiment (DoE) data set in pilot-scale bread making experiments. Error bars represent the confidence interval corresponding to a 95% confidence level. “*” between two processing factors indicates a synergistic effect, which cannot be explained solely by addition of degradation caused by the two parameters individually. Bottom: Predictive factor effect plots, which show the influence of the following processing parameters on the deoxynivalenol (DON) degradation during the biscuit production: (i) NaHCO3 concentration (left) and (ii) baking time (right). The dotted lines represent the confidence interval corresponding to a 95% confidence level.

For all three baking commodities, the results of the experimental trials were evaluated and presented in two sub-chapters. First, the sum of the DON degradation products, which is the measure for DON degradation, was shown as a bar graph. The individual experiments were listed according to the processing parameters that were found to be important for DON degradation based on a visual assessment. In this way, Figures 2, 4, and 6 show the DON degradation that was achieved under the specified processing conditions ranked from high to low DON degradation. These bar graphs give a first impression of the DON degradation and the influence of the processing parameter for the individual commodities. Second, to confirm and refine the conclusions of the visual assessment, a DoE model was built using the MODDE software tool. The outcome of this statistical analysis is visualized by an effects plot which displays the change of the sum of the DON degradation products when a processing factor is varied from its lowest to its highest value (Figures 3, 5, and 7, top). The data set obtained from the DoE model was used to build a prediction model for the influence of the process parameter that led to a statistically significant increase of DON degradation. These predictive factor plots show how DON degradation can be enhanced by the design of the production process (Figures 3, 5, and 9, bottom). To allow for the easy comparability of the results between the three baking commodities, the same scaling was chosen for the individual graphs.

#### 2.1.1. Biscuits

Biscuits, from the technological point of view, represent a quite simple bakery preparation characterized by a baking step and the use of raising agents. In order to monitor the impact of these two factors on the DON content in the final products, a very basic recipe was chosen, containing only flour, oil, sugar, and water, with the addition of NaHCO_3_ and NH_4_HCO_3_ as leavening agents. Several batches of biscuits were prepared at different conditions according to the DoE inputs based on the Screening Interaction model. In the present study, the processing parameters NaHCO_3_ concentration, NH_4_HCO_3_ concentration, baking time, baking temperature, and sugar content were varied (Table S1). Due to the different production conditions, DON degradation varied between 4–16% (Figure 2).

A higher DON degradation was observed in batches with a higher concentration of NaHCO_3_, baking temperature, and baking time. The variation of the processing parameter also led to a changed ratio of the degradation products with the main degradation products being norDON B and C, instead of isoDON, for experiments with a higher baking temperature and baking time. norDON A, which was not found to be a degradation product in our previous study [6], was found only at processing conditions that lead to higher DON degradation.

The DoE model confirmed that the main factors affecting DON degradation were: (i) The quantity of NaHCO_3_ used, (ii) the baking temperature, and (iii) the baking time (Figure 3, top). In addition, the statistical model indicates that a synergistic effect of baking temperature and time exists. Changes in NH_4_HCO_3_ and sucrose concentration did not significantly influence DON degradation products. The prediction model for the influence of the processing parameter on DON degradation revealed that by increasing the NaHCO_3_ concentration from 0.2 to 0.6%, DON degradation is increased by 5% (Figure 3, bottom). An increase in baking temperature and time is predicted to enhance DON degradation by about 2%.

#### 2.1.2. Bread

Bread is surely the most representative and most studied commodity of soft bakery products. Its production workflow implies dough fermentation before baking. Both fermentation and subsequent baking can be managed for time and temperature. The fermentation phase can also be modulated for relative humidity conditions, yeast, bakery improvers, raising agents, and pH. As a consequence, the number of factors to investigate for assessing potential DON mitigation effects was quite high. Therefore, in comparison to biscuits and crackers, more pilot plant trials had to be carried out. Based on literature data and our experience, the following variables were taken into account for the DoE approach: Baking temperature, baking time, leavening time, yeast, sucrose, and cider vinegar concentration. Each factor was varied within a range defined according to technological feasibility (Table S4). Depending on the processing parameter, DON degradation varied from 2–5% (Figure 4).

During the bread production, the achievable DON degradation was found to be low compared to the biscuit and cracker production. Although the influence was small, we found that the baking time and temperature as well as the sucrose concentration impacted the DON concentrations. The DoE model confirmed that the main factors influencing DON degradation were: (i) Baking time, (ii) baking temperature, and (iii) sucrose concentration (Figure 5, top). Changes in cider vinegar and yeast concentration as well as the leavening time did not influence DON degradation. The prediction model confirmed that changes in the processing parameter have only a minor effect of approximately 2% on DON degradation (Figure 5, bottom).

#### 2.1.3. Crackers

Crackers were chosen as being representative of a complex baking commodity involving different technological aspects and combining several factors which were demonstrated to affect DON mitigation in the previous experiments carried out on biscuits and bread. Among the cracker making parameters, the following conditions were considered as factors in the experimental design: Baking time, baking temperature, acidic mother content, and NaHCO_3_ concentration. For this trial, each factor was varied within a range defined according to technological feasibility (Table S7). During the cracker production, the DON degradation varied from 0–21%, depending on the choice of processing parameters (Figure 6).

A high DON degradation was observed in experiments with a high NaHCO_3_ concentration, baking time, and baking temperature. Similar to the biscuit production, the variation of the processing parameter caused a change in the ratio of the degradation products. The DoE model revealed that NaHCO_3_ concentration and baking time were the main factors affecting DON degradation (Figure 7, top). In addition, the statistical model indicated that a synergistic effect of baking time and NaHCO_3_ concentration exists. The acidic mother concentration, leavening time, and baking temperature did not affect DON degradation. The prediction model revealed the potential DON reduction that can be achieved by process management (Figure 7, bottom). An increase of the NaHCO_3_ concentration from 0 to 1% is predicted to increase DON degradation by 10%. Similarly, the increase of baking time from 1 to 6 min is predicted to increase DON degradation by 10%.

### 2.2. Cytotoxic Effects of isoDON

**Figure 8:**
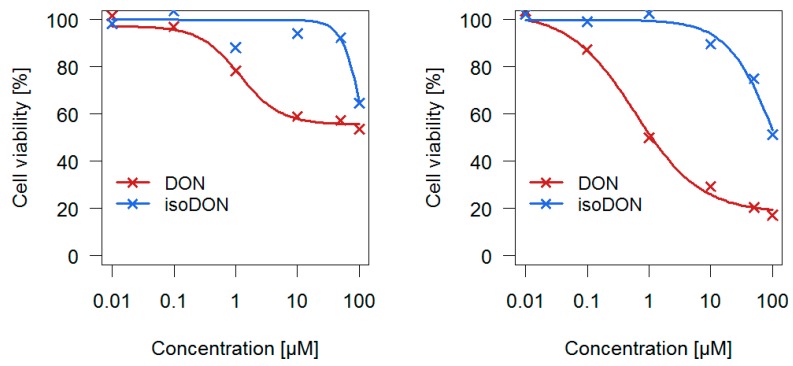
Cell viability of human colorectal adenocarcinoma cells (HT-29) (left) and non-tumorigenic human colon epithelial cells (HCEC) (right) after 24 h of incubation with different concentrations of deoxynivalenol (DON) and isoDON. The concentration at which cell viability was reduced by 30% (IC_30_) was calculated from a dose response curve fitted to the individual data points (×).

The cytotoxic potential of isoDON in direct comparison to DON was evaluated in two different human colon cell lines (Figure 8).

Usually, the half maximal inhibitory concentration (IC_50_) is used to compare the potency of two substances. As the highest tested isoDON concentrations did not reduce cell viability by 50% under all tested conditions, IC_30_ values where cell viability was reduced by 30% were used instead. For the human colorectal adenocarcinoma cell line HT-29 the following IC_30_ values were determined: 1.9 µM (DON) and 91 µM (isoDON), whereas for the non-tumorigenic human colon epithelial cells (HCEC) the following IC_30_ values were identified: 0.4 µM (DON) and 53 µM (isoDON). In both cell viability assays, isoDON was much less potent (factor 48 and 133 for HT-29 and HCEC, respectively) in reducing cellular viability compared to DON.

## 3. Discussion

### 3.1. Influence of Different Processing Parameters on DON Degradation in Bakery Products

#### 3.1.1. The pH Value of the Dough

As DON has been reported to be unstable in alkaline solutions, the pH of the dough is clearly an important processing factor to consider when designing a production process that is optimized for a high DON degradation [13]. For the production of biscuits and crackers, the pH of the dough was regulated primarily by chemical raising agents. We found that the type of the chemical raising agent as well as its concentration was crucial regarding DON degradation. Whereas the use of NaHCO_3_ led to higher DON degradation, the use of NH_4_HCO_3_ did not influence DON concentration. This can be explained by the different pH values of the dough resulting from the different chemical nature of the raising agents. Gökmen et al. prepared cookies from two different doughs containing 0.7% NaHCO_3_ and NH_4_HCO_3_, respectively [14]. When NaHCO_3_ was used, the pH was initially 8.5 and increased slightly during baking to pH 9. In the absence of acidic compounds, NaHCO_3_ is thermally converted to Na_2_CO_3_, H_2_O and CO_2_ (Equation (1)). As CO_3_^2−^ is a stronger base than HCO_3_^−^, the thermal degradation of NaHCO_3_ leads to an increase of the ph. The use of NH_4_HCO_3_ as a leavening agent led to a decrease of the pH from 8 in the dough to 6–7 in the baked cookies. The decrease of pH can be explained by the degradation of NH_4_HCO_3_ to NH_3,_ CO_2_ and H_2_O, which evaporate during baking (Equation (2)).

2 NaHCO_3_ → Na_2_CO_3_ + H_2_O + CO_2_ (1)

NH_4_HCO_3_ → NH_3_ + H_2_O + CO_2_ (2)

Besides chemical raising agents, further ingredients were shown to affect the pH of the dough. High pH values due to the presence of NaHCO_3_ were only observed when sucrose was used as sugar. At a higher pH, sucrose was not hydrolyzed and thereby did not cause a change in pH [14]. When glucose was used, it was partially hydrolyzed to fructose resulting in a decrease of the pH from 8.5 in the dough to 6–7 in the baked cookies. This can be rationalized by the report of Feng et al. who calculated the pKa values for glucose and fructose [15]. Fructose was shown to be considerably more acidic (pK_a_ 12) compared to glucose (pK_a_ 14). In the presence of an acid, NaHCO_3_ was converted to CO_2_ which caused a decrease of the pH (Equation (3)).

NaHCO_3_ + H^+^ → Na^+^ + H_2_O + CO_2_ (3)

For the biscuit production, our results confirm the outcome of a previous study, where the NaHCO_3_ concentration was the main factor that enhanced DON degradation [9]. For the cracker production, our results contradict a previous report in which baking temperature and time were found to be of higher importance compared to the NaHCO_3_ concentration. However, in the previous study, fewer experimental trials were carried out and the accuracy of the analytical methodology was lower.

#### 3.1.2. Baking Conditions

For all three commodities, the baking conditions (i.e., temperature and/or time) influenced DON degradation. However, for the production of biscuits and crackers, baking conditions were less important compared to the pH of the dough. For the production of crackers and bread, baking time was found to be more important than baking temperature. The contrary was observed for biscuit production. The reason for this observation might relate to differences in moisture content and surface to volume ratio of the individual baking commodities.

#### 3.1.3. Ratio of the DON Degradation Products

For the production of biscuits and crackers, the ratio of the DON degradation products shifted from isoDON to norDON B and C in the experiments that led to high DON degradation. This supports the proposed mechanism, that isoDON is further converted to norDON B and C [13]. Some baking conditions led to the formation of norDON A. Most likely, the reason why norDON A was not detected in a previous study was a higher limit of detection (LOD) and limit of quantification (LOQ) for norDON A compared to isoDON, norDON B and C in the analytical method used [6]. Interestingly, the formation of norDON A was not observed in the experiments with the highest DON degradation (i.e., biscuits: N4, N16; crackers: N11, N12, N15, N16) in total. This might have been due to an initial formation of isoDON, which was further degraded into norDON A and finally to norDON B or norDON C.

### 3.2. Toxicity of the DON Degradation Products

For a comprehensive assessment of the health risks of eating bakery products produced from DON contaminated flour, the toxicity of all degradation products has to be determined. So far, norDONs A–C were shown to be considerably less cytotoxic than DON [5]. In comparison to DON, isoDON was found in in vitro translation assays to have a considerably lower potency (factor 94 and 60 in wheat and rabbit ribosomes, respectively) to inhibit translation, known to be the major mechanism of toxicity of trichothecenes [6]. As the 60S subunit binding site of ribosomes is the main molecular target of DON, the results of the translation assay were indicative of considerably lower cytotoxicity [16,17]. However, various aspects, such as different cellular absorption, changes in distribution, and especially differences in fitting into the pockets of the ribosomal A-site due to changes in polarity might lead to significant differences in cytotoxicity of isoDON and DON. Therefore, the cytotoxic effects of isoDON in comparison to DON were evaluated.

#### Cytotoxic Effects of isoDON

**Figure 9:**
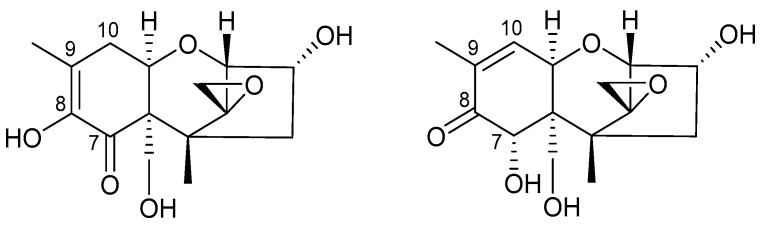
Structure of iso-deoxynivalenol (isoDON) (left) and DON (right). The C-7 to C-10 atoms of the trichothecene backbone are highlighted.

Performing cell viability assays in two different human colon cell lines, we found that isoDON induced significantly less cytotoxicity compared to DON in HCEC and HT-29 cells. In Figure 9, the structures of DON and isoDON, showing structural differences at the carbon atoms C-7 to C-10 of the trichothecene backbone, are displayed.

Our findings are supported by structure activity relationships which have been conducted for various trichothecenes [18,19,20]. The absence of a double bond at the C-9 and C-10 position, which is the case for isoDON, led to a significant decrease in both inhibition of protein biosynthesis and cytotoxicity. Although the toxicity of DON is often ascribed to the epoxide moiety, we could provide additional proof that a double bond between C-9 and C-10 is an essential structural feature for ribosomal inhibition and thus, for trichothecene-induced toxicity.

## 4. Conclusions

Depending on the processing conditions, 0–21%, 4–16%, and 2–5% of DON were degraded during the production of crackers, biscuits, and bread, respectively. For biscuits and crackers, DON degradation can be increased by 10–20% by process management. For bread, process management can lead to a minor increase in DON degradation of about 3%. DON degradation was enhanced by a high NaHCO_3_ concentration, baking time, and temperature. The processing parameters NH_4_HCO_3_, yeast, cider vinegar, acidic mother content, and sucrose concentration as well as leavening time did not influence DON concentration.

**Figure 10:**
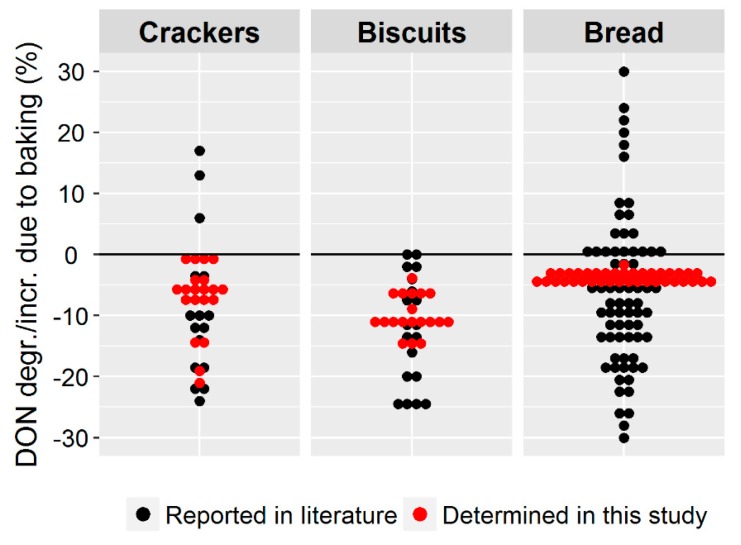
Comparison between the deoxynivalenol (DON) degradation reported in recent literature studies [10,11,12,21,22,23,24,25,26,27,28,29] and the DON degradation values determined in this study under different processing conditions. Each dot represents one baking experiment.

To put our findings in context to the results reported in recent literature, a graphical summary is presented in Figure 10.

Compared to previous studies found in the literature, we achieved a much narrower range of DON degradation, which was most pronounced for bread. The reason for that is most likely that we applied the currently most accurate analytical methodology to determine DON degradation [6].

To gain further knowledge about the toxicity of DON degradation products, the cytotoxicity of isoDON was evaluated by performing in vitro cell viability assays. isoDON was found to be considerably less cytotoxic (factor 48 and 133 for HT-29 and HCEC cells, respectively) compared to DON. As all DON degradation products are considerably less cytotoxic than DON, we conclude that DON degradation during baking results in a lower toxicity. Thus, for the first time, we have presented proof that baking under real industrial conditions can lead to a partial detoxification of DON.

## 5. Materials and Methods

### 5.1. Chemicals and Reagents

Acetonitrile (ACN, gradient grade) was purchased from VWR International GmbH (Vienna, Austria). Acetic acid (LC–MS gradient grade) was obtained from Sigma Aldrich (Vienna, Austria). In all experiments, ultra-pure water (purified by a Purelab Ultra system ELGA LabWater, Celle, Germany) was used. Liquid calibrant solutions of DOM-1, DON-3-Glc, DON, and U-[^13^C_15_]-DON were supplied by Romer Labs GmbH (Tulln, Austria). Reference standards of the DON degradation products isoDON and norDONs A, B and C were synthesized according to published procedures [5,6,21,30].

### 5.2. Preparation of Bakery Products

The food commodities used in the present study were produced from naturally contaminated flour according to the procedure previously described by our group by applying a suitable scaling up factor of the starting ingredients that fits with the production under pilot plant facilities [6].

Biscuits: The final dough (3000 g, 14% moisture content (MC)) was used to form the individual biscuits (approximately 11 g each) which consisted of 56 wt % flour, 40 wt % fat and sugar, and 4 wt % water.

Bread: The final dough (8000 g, 14% MC) was used to form the individual loafs of 450 g each (40% MC), that reached a final weight of 400 g and consisted of 65 wt % flour, yeast, oil, and salt and 35 wt % water after baking.

Crackers were shaped (approximately 10 g each) from the final dough (4127 g, 26% MC) and they consisted of 97 wt % flour, yeast, and malt extract and 3 wt % water.

### 5.3. Design of Experiment (DoE) Setup and Statistical Evaluation

Design of Experiments (DoE) was used to determine the influence of processing parameters on the degradation of DON and the increase of its degradation products during the baking of crackers, biscuits, and bread from naturally contaminated flour. The screening interaction model was chosen and processing parameters that we hypothesized to influence DON degradation were used as model quantitative factors. Table 1 provides a list of the processing parameters that were investigated in our study. For more detailed information on the processing parameters used in the individual experiments, the reader is referred to Tables S1, S4, and S7.

To focus the attention on the increase of DON degradation products during baking, multivariate data analysis was performed on the concentration (µg/kg) obtained by LC/MS-MS analysis in the finished products of the sum of DON degradation products. DoE set up and data elaboration were carried out by using MODDE software 11 (Umetrics, Umea, Sweden).

### 5.4. Analysis of the Samples

To determine DON degradation during the industrial baking process, the concentrations of DON and its degradation products (i.e., isoDON, norDON B, and norDON C) were analyzed in naturally contaminated flour and the products made thereof. Additionally, the concentration of the suspected DON degradation and conversion products DON-3-glucoside, DOM-1 and norDON A was monitored. Sample preparation and analysis are discussed in detail elsewhere [6]. In brief, 5.00 ± 0.01 g of ground and homogenized sample was extracted with 20.0 mL of ACN:H_2_O, 84:16, *v*:*v*. A total of 200 µL of sample extract were dried down and reconstituted in 100 µL of water. Subsequently, the concentrated sample extracts were centrifuged at 10 °C and 4500 rpm for 10 min. Four µL aliquots of the concentrated sample extract were injected together with 0.4 µL of internal standard into the HPLC system. Each sample was worked-up once and injected in duplicate. The analytical method used for the analysis was based on LC-MS/MS and was validated for recovery, repeatability, LOD, and LOQ. All results were corrected for the recovery factor determined in the validation study [6]. The concentration of the analytes that was found in naturally contaminated flour and in the finished bakery products can be found in Table S2 and S3 (biscuits), Table S5 and S6 (bread), and Table S8 and S9 (crackers) of the Supplementary Material, respectively.

### 5.5. Calculation of the DON Degradation

The sum of the DON degradation products was shown to be a much more accurate measure of the DON degradation than the change of the DON content itself [6]. Therefore, the DON degradation was calculated based on the increase of its degradation products isoDON and norDONs A–C. As the flour used for the production of the bakery products was naturally contaminated with the degradation products at different concentration levels, the increase of the degradation products was calculated separately before being summed up to obtain the final DON degradation.

### 5.6. Calculation of the Increase of the DON Degradation Products Due to Baking

For the production of bakery products from naturally contaminated flour, the increase of the degradation products was calculated according to:

$$dilution\;factor=\frac{m_{flour}}{m_{bakery\;product}}$$

$$b_{analyte,dilution\;of\;nat.cont.\;flour}=b_{analyte,\;flour}*dilution\;factor$$

$$Change\;due\;to\;baking=\frac{b_{analyte,\;bakery\;product}-b_{analyte,dilution\;of\;nat.cont.\;flour}}{b_{DON,dilution\;of\;nat.cont.\;flour}}$$

First, the dilution factor, which describes the mass ratio of naturally contaminated flour present in the finished bakery product, was determined. The dilution factor was 0.97 (crackers), 0.56 (biscuits), and 0.65 (bread), respectively. Second, the theoretical molal concentration b (mol/kg) of the analyte resulting from the dilution of the flour by the addition of other ingredients ($b_{analyte,dilution\;of\;nat.cont.\;flour}$) was determined. Finally, the molal concentration of an analyte resulting from the natural contamination of the flour was subtracted from the molal concentration of the analyte in the bakery product ($b_{analyte,\;bakery\;product}$) and expressed as a fraction of the molal concentration of DON resulting from the natural contamination of the flour. The result was the fraction of DON that is converted to DON degradation products during the production of bakery products.

### 5.7. Estimation of the Process Standard Deviation

In each DoE, three center points were set, which means that three experiments were carried out under identical processing conditions: Experiment number 17, 18 and 19 (crackers), 17, 18 and 19 (biscuits) and 33, 34, 35 (bread), respectively. The process standard deviation was calculated for the sum of the change of the degradation products of the three replicates which were produced using the same processing parameter. The process standard deviation was 0.9%, 1.4%, and 0.8% for crackers, biscuits, and bread, respectively, and thus considerably higher than the analytical standard deviation for the determination of the sum of the DON degradation products which was in the range of 0.2–0.4% [6].

### 5.8. Human Cell Culture

Human colorectal adenocarcinoma cells, HT-29, were purchased from the German Collection of Microorganisms and Cell Cultures (DSMZ, Braunschweig, Germany). For cell culture Dulbecco’s Modified Eagle’s Medium (DMEM) supplemented with 10% (*v*/*v*) heat inactivated fetal calf serum and 50 U/mL penicillin and 50 µg/mL streptomycin was used. The non-tumorigenic human colon epithelial cell line, HCEC [31], was kindly provided by Prof. Jerry W. Shay (UT Southwestern Medical Center, Dallas, TX, USA). HCEC cells were cultivated in DMEM (high glucose) supplemented with 2% (*v*/*v*) Medium 199 (10×), 2% (*v*/*v*) cosmic calf serum, 20 mM 4-(2-hydroxyethyl)-1-piperazineethanesulfonic acid (HEPES), 50 µg/mL gentamicin, insulin-transferrin-selenium-G (10 µg/mL; 5.5 µg/mL; 6.7 µg/mL), recombinant human epidermal growth factor (18.66 ng/mL), and hydrocortisone (1 µg/mL). Both cell lines were cultivated in humidified incubators at 37 °C and 5% CO_2_. Media, supplements, and further material for cell culture were purchased from GIBCO Invitrogen (Karlsruhe, Germany), Lonza Group Ltd. (Basel, Switzerland), Sigma-Aldrich Chemie GmbH (Munich, Germany), Sarstedt AG & Co. (Nuembrecht, Germany), and Fisher Scientific GmbH (Vienna, Austria).

### 5.9. Cytotoxicity of isoDON

Per well, 1500 HCEC or 5500 HT-29 cells were seeded in a 200 μL culture medium in 96-well plates and allowed to grow for 48 h. Then, the culture medium was withdrawn and cells were treated with different concentrations of isoDON and DON in the respective cell culture medium for 24 h. As a solvent control, 1% water (LC–MS grade) was incubated and 1% (*v*/*v*) Triton X-100 served as a positive control. After the treatment period, the medium was withdrawn and cells were rinsed once with phosphate buffered saline (100 μL/well) and incubated with DMEM containing 10% (*v*/*v*) alamarBlue^®^ reagent (Invitrogen™ Life Technologies, Karlsruhe, Germany) for 75 min. Subsequently, fluorescence intensity was measured (excitation: 530 nm; emission: 600 nm) on a Cytation 3 Imaging Multi Mode Reader (BioTek, Bad Friedrichshall, Germany). For quantification, blank-values were subtracted and measured data were referred to solvent control. Data are presented as a mean of at least five independent experiments, each performed in technical triplicates.

## Figures and Tables

**Table 1:** Processing parameters that were investigated for their impact on the degradation of deoxynivalenol during the production of bakery products from naturally contaminated flour under pilot plant conditions.

Condition	Biscuits	Bread	Crackers
Sucrose concentration [%]	12.5 to 17.5	0.41 to 1.41	-
NH_4_HCO_3_ concentration [%]	0.21 to 0.61	-	-
NaHCO_3_ concentration [%]	0.19 to 0.59	-	0.00 to 0.96
Vinegar concentration [%]	-	0.00 to 0.36	-
Yeast concentration [%]	-	0.83 to 1.33	-
Leavening time [%]	-	70 to 100	-
Acidity Mother [%]	-	-	0.65 to 1.65
Baking time [min]	7 to 11	15 to 29	1 to 6
Baking temperature [°C]	160 to 200	185 to 225	230 to 250
Total experiments	19	38	20

## Supplementary Materials

The following are available online at https://www.mdpi.com/2072-6651/11/6/317/s1, Table S1: Processing parameters used for the production of biscuits in different experiments (Exp. No.). Ingredients are given as the weight percentage of the dough; Table S2: Concentration of the analytes in the naturally contaminated flour, dilution factor by which the flour gets diluted in the final biscuits, and the resulting concentration (assuming 0% degradation). <LOQ: concentration was below the limit of quantification (LOQ) of the analytical methodology. Table S3: Concentration of deoxynivalenol (DON) and related compounds in the biscuit samples. <LOQ: concentration was below the limit of quantification (LOQ) of the analytical methodology; Table S4: Processing parameters used for the production of bread in different experiments (Exp. No.). Ingredients are given as the weight percentage of the dough; Table S5: Concentration of the analytes in the naturally contaminated flour, dilution factor by which the flour gets diluted in the final bread, and the resulting concentration (assuming 0% degradation). n.d. (not detected): Concentration was below the limit of quantification of the analytical methodology; Table S6: Concentration of deoxynivalenol (DON) and related compounds in bread samples. n.d. (not detected): Concentration was below the limit of quantification of the analytical methodology; Table S7: Processing parameters used for the production of crackers in different experiments (Exp. No.). Ingredients are given as the weight percentage of the dough; Table S8: Concentration of the analytes in the naturally contaminated flour, dilution factor by which the flour gets diluted in the final crackers, and the resulting concentration (assuming 0% degradation). n.d. (not detected): Concentration was below the limit of quantification of the analytical methodology; Table S9: Concentration of deoxynivalenol (DON) and related compounds in cracker samples. n.d. (not detected): Concentration was below the limit of quantification of the analytical methodology.
