# Supplementary material for: The Influence of Processing Parameters on the Mitigation of Deoxynivalenol during Industrial Baking
Source: Toxins (Basel). 2019 Jun 4;11(6):317. doi: 10.3390/toxins11060317 (PMC6628453; doi:10.3390/toxins11060317)
Supplement: Supplementary file 1 [file toxins-11-00317-s001.pdf]

Supplementary Materials: The influence of processing parameters on the mitigation of deoxynivalenol during industrial baking

David Stadler, Francesca Lambertini, Lydia Woelflingseder, Heidi Schwartz-Zimmermann, Doris Marko, Michele Suman, Franz Berthiller and Rudolf Krska

Design of Experiment Study, Biscuits

Table 1. Processing parameters used for the production of biscuits in different experiments (Exp. No.). Ingredients are given as the weight percentage of the dough.

Exp. No.	Baking temp. (°C)	Baking time (min)	Sucrose (%)	NH_4HCO_3 (%)	$NaHCO_3$ (%)
1	160	7	12.5	0.21	0.59
2	200	7	12.5	0.21	0.19
3	160	11	12.5	0.21	0.19
4	200	11	12.5	0.21	0.59
5	160	7	17.5	0.21	0.19
6	200	7	17.5	0.21	0.59
7	160	11	17.5	0.21	0.59
8	200	11	17.5	0.21	0.19
9	160	7	12.5	0.61	0.19
10	200	7	12.5	0.61	0.59
11	160	11	12.5	0.61	0.59
12	200	11	12.5	0.61	0.19
13	160	7	17.5	0.61	0.59
14	200	7	17.5	0.61	0.19
15	160	11	17.5	0.61	0.19
16	200	11	17.5	0.61	0.59
17	180	9	15.0	0.41	0.39
18	180	9	15.0	0.41	0.39
19	180	9	15.0	0.41	0.39

Table 2. Concentration of the analytes in the naturally contaminated flour, dilution factor by which the flour gets diluted in the final biscuits, and the resulting concentration (assuming 0 % degradation). <LOQ: concentration was below the limit of quantification (LOQ) of the analytical methodology.

Analyte	Flour (µg/kg)	Dilution factor	Biscuits theoretical (µg/kg)
DON-3-Glc	110	0.56	62
DON	1741	0.56	975
isoDON	114	0.56	64
DOM-1	3.6	0.56	2.0
norDON A	< LOQ	0.56	-
norDON B	7.0	0.56	3.9
norDON C	6.8	0.56	3.8

Table 3. Concentration of deoxynivalenol (DON) and related compounds in the biscuit samples. <LOQ: concentration was below the limit of quantification (LOQ) of the analytical methodology.

Exp. No.	DON (µg/kg)	DON-3-Glc (µg/kg)	isoDON (µg/kg)	DOM-1 (µg/kg)	norDON A (µg/kg)	norDON B (µg/kg)	norDON C (µg/kg)
1	925	101	131	2.6	11.6	19.2	24.0
2	1020	110	98	2.8	<LOQ	13.6	15.5
3	1012	107	101	2.5	<LOQ	11.9	13.7
4	872	78	123	3.5	<LOQ	44.6	53.7
5	973	93	90	2.6	<LOQ	9.0	10.1
6	900	83	115	3.4	9.9	23.8	29.7
7	892	86	115	2.6	11.5	20.7	25.7
8	898	81	121	3.1	<LOQ	26.8	27.6
9	1017	110	107	3.3	<LOQ	10.6	11.7
10	971	95	131	2.9	17.5	25.6	32.4
11	1015	100	123	4.1	12.3	21.5	27.0
12	1036	95	128	3.3	<LOQ	22.7	24.9
13	880	89	133	2.7	8.3	18.4	23.3
14	976	93	108	3.6	<LOQ	14.2	16.8
15	956	95	111	2.3	<LOQ	13.6	15.5
16	753	62	100	2.7	<LOQ	50.5	62.4
17	982	88	118	3.4	7.8	21.2	25.5
18	997	93	113	3.1	6.4	17.5	21.9
19	927	90	116	2.9	10.2	26.9	32.9

Design of Experiment Study, Bread

Table 4. Processing parameters used for the production of bread in different experiments (Exp. No.). Ingredients are given as the weight percentage of the dough.

Exp. No.	Baking temp. (°C)	Baking time (min)	Vinegar (%)	Yeast (%)	Sucrose (%)	Leaving time (min)
N1	185	15	0	0.83	0.41	100
N2	225	15	0	0.83	0.41	70
N3	185	29	0	0.83	0.41	70
N4	225	29	0	0.83	0.41	100
N5	185	15	0.36	0.83	0.41	70
N6	225	15	0.36	0.83	0.41	100
N7	185	29	0.36	0.83	0.41	100
N8	225	29	0.36	0.83	0.41	70
N9	185	15	0	1.33	0.41	100
N10	225	15	0	1.33	0.41	70
N11	185	29	0	1.33	0.41	70
N12	225	29	0	1.33	0.41	100
N13	185	15	0.36	1.33	0.41	70
N14	225	15	0.36	1.33	0.41	100
N15	185	29	0.36	1.33	0.41	100
N16	225	29	0.36	1.33	0.41	70

N17	185	15	0	0.83	1.41	70
N18	225	15	0	0.83	1.41	100
N19	185	29	0	0.83	1.41	100
N20	225	29	0	0.83	1.41	70
N21	185	15	0.36	0.83	1.41	100
N22	225	15	0.36	0.83	1.41	70
N23	185	29	0.36	0.83	1.41	70
N24	225	29	0.36	0.83	1.41	100
N25	185	15	0	1.33	1.41	70
N26	225	15	0	1.33	1.41	100
N27	185	29	0	1.33	1.41	100
N28	225	29	0	1.33	1.41	70
N29	185	15	0.36	1.33	1.41	100
N30	225	15	0.36	1.33	1.41	70
N31	185	29	0.36	1.33	1.41	70
N32	225	29	0.36	1.33	1.41	100
N33	205	22	0	1.08	0.91	70
N34	205	22	0	1.08	0.91	70
N35	205	22	0	1.08	0.91	70
N36	185	29	0	0.83	1.41	70
N37	185	15	0	0.83	1.41	100
N38	205	22	0.36	1.33	0.91	70

Table 5. Concentration of the analytes in the naturally contaminated flour, dilution factor by which the flour gets diluted in the final bread, and the resulting concentration (assuming 0 % degradation). n.d. (not detected): Concentration was below the limit of quantification of the analytical methodology.

Analyte	Flour (µg/kg)	Dilution factor	Bread theoretical (µg/kg)
DON-3-Glc	108	0.65	71
DON	1814	0.65	1179
isoDON	118	0.65	77
DOM-1	4.3	0.65	2.8
norDON A	n.d.	0.65	-
norDON B	7.3	0.65	4.8
norDON C	7.1	0.65	4.6

Table 6. Concentration of deoxynivalenol (DON) and related compounds in bread samples. n.d. (not detected): Concentration was below the limit of quantification of the analytical methodology.

Exp. No.	DON (µg/kg)	DON-3-Glc (µg/kg)	isoDON (µg/kg)	DOM-1 (µg/kg)	norDON A (µg/kg)	norDON B (µg/kg)	norDON C (µg/kg)
1	1272	73	107	3.7	n.d.	7.5	5.7
2	1248	65	110	3.6	n.d.	8.2	6.5
3	1209	54	109	3.2	n.d.	8.9	7.2
4	1144	45	106	2.7	n.d.	14.7	10.3
5	1114	63	95	3.0	n.d.	6.4	5.4
6	1281	55	114	3.2	n.d.	13.1	9.1

7	1240	54	110	3.1	n.d.	9.0	7.1
8	1260	66	114	3.1	n.d.	9.2	7.3
9	1194	67	103	3.1	n.d.	6.9	5.9
10	1226	63	106	2.8	n.d.	8.3	6.5
11	1262	55	113	3.2	n.d.	9.9	7.8
12	1280	47	115	3.0	n.d.	17.7	11.9
13	1208	61	103	3.0	n.d.	7.3	5.9
14	1185	79	106	2.8	n.d.	9.6	8.5
15	1208	49	110	2.6	n.d.	10.0	7.7
16	1349	78	120	3.1	n.d.	13.7	10.1
17	1192	45	113	3.3	n.d.	7.2	5.9
18	1240	61	112	3.1	n.d.	9.1	6.9
19	1298	39	127	3.3	n.d.	10.3	8.4
20	1253	43	117	2.9	n.d.	13.8	9.3
21	1244	62	108	3.1	n.d.	7.0	5.9
22	1215	43	113	3.7	n.d.	8.9	6.9
23	1259	53	115	3.1	n.d.	9.4	7.3
24	1233	32	116	3.2	n.d.	16.5	11.0
25	1312	67	108	3.0	n.d.	7.6	6.8
26	1302	62	119	2.8	n.d.	10.8	8.2
27	1435	56	125	3.1	n.d.	10.8	8.5
28	1291	48	122	3.0	n.d.	13.3	9.8
29	1216	62	107	2.6	n.d.	7.1	6.0
30	1274	82	117	3.3	n.d.	8.9	7.2
31	1241	52	114	3.2	n.d.	9.3	7.1
32	1334	72	119	2.3	n.d.	15.5	10.6
33	1434	61	127	3.4	n.d.	11.0	7.9
34	1249	56	111	3.1	n.d.	9.7	7.3
35	1303	62	119	3.5	n.d.	8.8	7.3
36	1282	53	117	3.3	n.d.	10.2	7.7
37	1240	71	104	3.4	n.d.	7.3	6.4
38	1221	42	112	3.4	n.d.	10.4	8.1

Design of Experiment Study, Crackers

Table 7. Processing parameters used for the production of crackers in different experiments (Exp. No.). Ingredients are given as the weight percentage of the dough.

Exp. No.	Baking temp. (°C)	Baking time (min)	ac. Mother (%)	NaHCO3 (%)
N1	230	1	0.65	0
N2	250	1	0.65	0
N3	230	6	0.65	0
N4	250	6	0.65	0
N5	230	1	1.65	0
N6	250	1	1.65	0
N7	230	6	1.65	0
N8	250	6	1.65	0

N9	230	1	0.65	0.96
N10	250	1	0.65	0.96
N11	230	6	0.65	0.96
N12	250	6	0.65	0.96
N13	230	1	1.65	0.96
N14	250	1	1.65	0.96
N15	230	6	1.65	0.96
N16	250	6	1.65	0.96
N17	240	3.5	1.15	0.48
N18	240	3.5	1.15	0.48
N19	240	3.5	1.15	0.48
N20	250	3	1.15	0.48

Table 8. Concentration of the analytes in the naturally contaminated flour, dilution factor by which the flour gets diluted in the final crackers, and the resulting concentration (assuming 0 % degradation). n.d. (not detected): Concentration was below the limit of quantification of the analytical methodology.

Analyte	Flour (µg/kg)	Dilution factor	Crackers theoretical (µg/kg)
DON-3-Glc	92	0.97	90
DON	1409	0.97	1367
isoDON	98	0.97	95
DOM-1	3.6	0.97	3.5
norDON A	n.d.	0.97	-
norDON B	6.2	0.97	6.0
norDON C	7.5	0.97	7.3

Table 9. Concentration of deoxynivalenol (DON) and related compounds in cracker samples. n.d. (not detected): Concentration was below the limit of quantification of the analytical methodology.

Exp. No.	DON (µg/kg)	DON-3-Glc (µg/kg)	isoDON (µg/kg)	DOM-1 (µg/kg)	norDON A (µg/kg)	norDON B (µg/kg)	norDON C (µg/kg)
1	1279	78	106	4.1	n.d.	7.5	8.5
2	1320	73	109	4.2	n.d.	7.5	8.4
3	1410	52	144	3.7	n.d.	20.9	14.6
4	1152	26	131	3.2	n.d.	34.2	24.2
5	1275	70	105	3.3	n.d.	7.2	8.0
6	1218	72	102	3.5	n.d.	7.3	8.3
7	1390	54	133	3.4	n.d.	17.4	12.6
8	1429	56	137	4.0	n.d.	18.6	14.4
9	1208	85	148	3.7	16.3	17.0	22.7
10	1211	86	150	3.4	19.8	19.1	25.3
11	1096	43	122	2.5	n.d.	79.6	79.6
12	691	18	74	1.9	n.d.	154.9	137.7
13	1242	74	145	3.2	12.5	16.0	19.4
14	1156	69	142	2.6	17.7	19.4	24.9
15	1066	42	117	2.2	n.d.	89.8	92.2
16	873	26	97	1.4	n.d.	135.7	113.1

17	1423	77	145	4.0	n.d.	16.9	18.2
18	1398	77	141	3.9	n.d.	19.0	19.7
19	1338	59	145	2.7	n.d.	30.0	29.0
20	1377	69	140	3.1	n.d.	20.9	20.8
